# Intramucosal Signet Ring Cell Gastric Cancer Diagnosed 15 Months after the Initial Endoscopic Examination

**DOI:** 10.1155/2015/479625

**Published:** 2015-07-06

**Authors:** Keisuke Taniuchi, Koji Ookawauchi, Kento Kumon, Tatsuaki Sumiyoshi, Jun Iwata, Mutsuo Furihata, Toshio Nakamura, Junko Uchiumi

**Affiliations:** ^1^Department of Gastroenterology, Shinmatsudakai Atago Hospital, Kochi 780-0051, Japan; ^2^Department of Endoscopic Diagnostics and Therapeutics, Kochi Medical School, Kochi University, Nankoku 783-8505, Japan; ^3^Department of Gastroenterological Surgery, Kochi Health Sciences Center, Kochi 781-0111, Japan; ^4^Department of Diagnostic Pathology, Kochi Health Sciences Center, Kochi 781-0111, Japan; ^5^Department of Pathology, Kochi Medical School, Kochi University, Nankoku 783-8505, Japan

## Abstract

The size and shape of intramucosal signet ring gastric cancer in this case remained endoscopically unchanged for 15 months. Laparoscopy-assisted distal gastrectomy was performed, and immunohistochemical analysis revealed Ki-67 and p53 mutations to be negative in this case. Signet ring gastric cancer has long been thought to confer a worse prognosis than other forms of gastric cancer; however, our case did not progress to advanced gastric cancer for 15 months.

## 1. Introduction

Signet ring cell gastric cancer (SRGC) is a histologic diagnosis based on microscopic characteristics as described by the World Health Organization [1]. SRGC cells are characterized by abundant intracytoplasmic mucins, ample and clear cytoplasm, and eccentrically located nuclei compressed by intracytoplasmic mucins [2]. The clinicopathological characteristics of SRGC are known to differ from other types of gastric cancer. Some reports have suggested a higher rate of formation of multiple gastric cancers if the primary lesion is SRGC [3, 4].

In general, SRGC is believed to behave more aggressively and have a worse prognosis than other forms of gastric cancer [5]; however, recent studies from Asia have demonstrated that when adjusted for stage, this might not be the case [6]. Early SRGC does not demonstrate more frequent lymph node (LN) metastases than other types of gastric cancer; improved survival has been reported for early stages of SRGC compared with other types of gastric cancer [7], but relatively worse survival in later stages of the disease [8]. Once early SRGC evolves into advanced gastric cancer, the diffusely infiltrating characteristics of SRGC may involve the entire stomach, resulting in what is known as linitis plastica, which is associated with a poor prognosis [4]. Thus, the idea that signet ring cell histology conveys a worse prognosis might not always be correct.

Here, we report a rare case of SRGC in a male patient whose flat pale mucosal lesion was endoscopically unchanged 15 months after the initial endoscopic examination, when he was diagnosed with intramucosal SRGC.

## 2. Case Report

**Figure 1:**
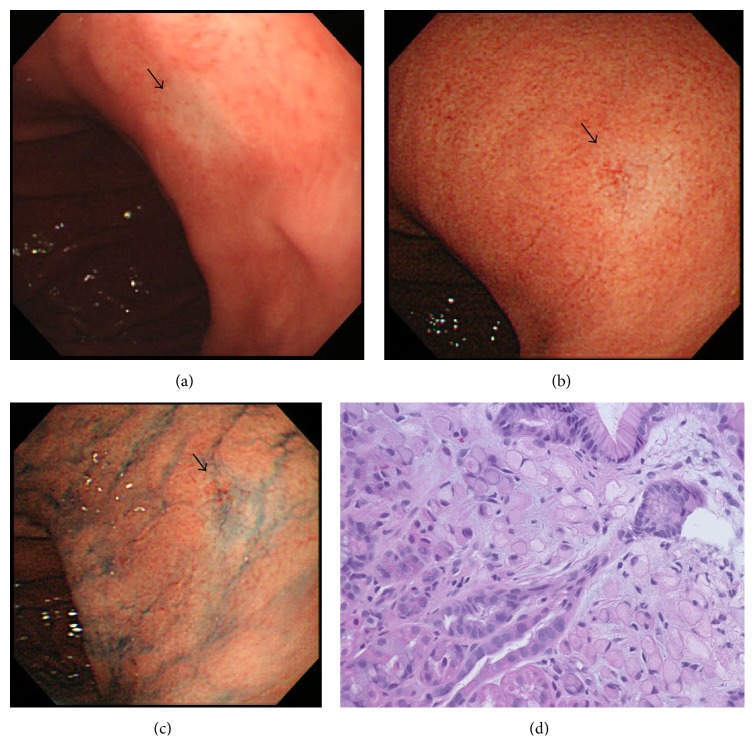
Endoscopic examination before treatment. Conventional white-light endoscopy in October 2011, when the initial examination was carried out ((a) arrow: SRGC lesion). Conventional white-light endoscopy in January 2013, when initial diagnosis was made ((b) arrow: SRGC lesion), and with indigo-carmine dye staining added to acetic acid in January 2013 ((c) arrow: SRGC lesion). The biopsy specimen revealed signet ring cells ((d) ×40).

**Figure 2:**
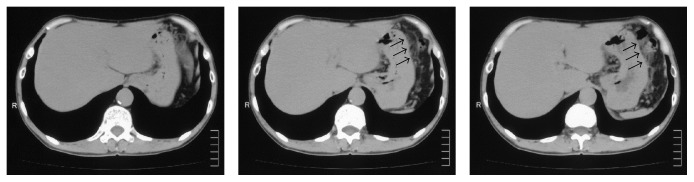
Abdominal computed tomography before treatment. Computed tomography showed fluffing surrounding the gastric body (arrows).

**Figure 3:**
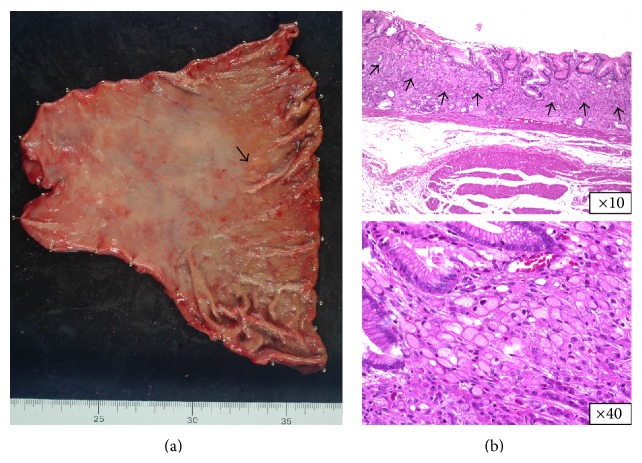
The surgically resected specimen and histopathological findings. Resected specimen from laparoscopy-assisted distal gastrectomy ((a) arrow: SRGC lesion). The tumor cells showed signet ring cell components (low magnification view ×10); high magnification (high magnification view ×40) of signet ring cell components ((b) arrows: signet ring cells limited to the mucosa).

**Figure 4:**
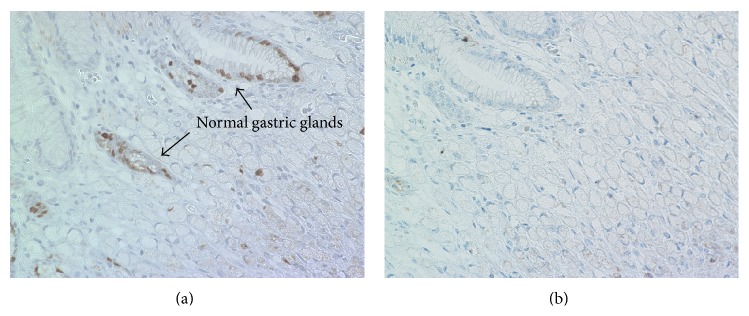
Immunohistochemical staining. Tumor cells were negative for Ki-67 ((a) ×40) and p53 ((b) ×40).

A 58-year-old man who had suffered from appetite loss underwent an esophagogastroduodenoscopy (EGD) during a medical check-up in October 2011. A flat, pale, mucosal lesion (5 mm in diameter; Figure 1(a)) on the posterior wall of the distal gastric body was observed. Chromoendoscopy with indigo-carmine dye added to acetic acid did not show any staining indicating gastric neoplasia, so we planned to repeat EGD 1 year later. The herbal medicine* rikkunshito* was administered for 4 weeks, and the patient's appetite gradually improved. The next EGD to which the patient consented, taken in January 2013, revealed the flat, pale, mucosal lesion to be the same size as it had been in October 2011 (Figure 1(b)); it was not enhanced by indigo-carmine dye staining added to acetic acid (Figure 1(c)). Additionally, image-enhanced EGD using the narrow-band light method did not reveal any findings of significance (data not shown). At this time, we took a biopsy to pathologically diagnose and characterize this gastric lesion, which allowed diagnosis of SRGC (Figure 1(d)). The endoscopic diagnosis was Type 0-IIb in accordance with the Japanese Classification of Gastric Carcinoma (JCGC) [9], which is a manual that describes the classification of cancer according to its stage and histology, classifies its curability via surgery and the extent of lymphadenectomy, and evaluates responses to chemotherapy [10]. Computed tomography (CT) showed fluffing surrounding the gastric body and no distinct regional LN or distant metastases (Figure 2). The fluffing indicated inflammatory changes suspected to be serosal invasion from the gastric tumor. Laboratory results were within normal limits and the serum carcinoembryonic antigen level was normal. We diagnosed this lesion as clinical(c) T1(M), cN0, cM0, and cStage IA according to the JCGC. The CT finding of inflammatory changes surrounding the gastric body suggested a risk of serosal invasion, so laparoscopy-assisted distal gastrectomy was performed rather than endoscopic resection. The regional lymph nodes (LN stations 1, 3, 4d, 5, 6, 7, 8a, 9, and 12a) were resected. The surgical specimen revealed signet ring cells alone, limited to the mucosa without lymphovascular involvement or ulcerative findings (Figures 3(a)-3(b)). The lesion was 5 mm in diameter, and the resection margin was free of tumor cells. Portions of the resected regional LNs did not include metastatic cancer cells. Thus, this lesion was diagnosed as pathological (p)T1(M), ly0, v0, pN0, pM0, R0, and pStage IA according to the JCGC [9]. In addition, immunohistochemical analysis showed that tumor cells were negative for both Ki-67 (Figure 4(a)) and p53 (Figure 4(b)). Eleven months after surgery, the patient was in good condition and disease-free without any signs of tumor recurrence based on the latest follow-up EGD and CT examinations.

## 3. Discussion

Early SRGC tends to be superficial and large, so it is usually diagnosed earlier than gastric cancer of other histological types [3]. At the first endoscopy (October 2011), we could detect a small, flat, pale mucosa in the stomach. Chromoendoscopy with indigo-carmine dye added to acetic acid improves the detection rates of early gastric cancer and precancerous lesions [11]. In this case, indigo-carmine dye added to acetic acid staining did not show any signs indicating gastric neoplasia, so we did not take any biopsies in the initial EGD. Therefore, it is unknown whether signet ring cells had been histologically localized in this gastric lesion in the initial EGD. The next endoscopic images taken in January 2013 showed that the lesion was unaltered in size and shape; satellite lesions were not seen, and biopsy was carried out at this time. Unexpectedly, the biopsy revealed SRGC histology. It is unknown whether SRGC usually arises from a preexisting precancerous lesion or as a so-called de novo carcinoma [5]. Conventional endoscopy and chromoendoscopy did not show any alterations in size or shape of this gastric lesion for 15 months between the first and second examinations, and this tumor showed only signet ring cells without accompanying adenoma or other types of cancer cells. Thus, there was a low possibility that a preexistent precancerous lesion had turned into SRGC in the intramucosal layer between the first and second examinations; therefore, it is likely that signet ring cells might have been localized in this gastric lesion in the initial EGD.

In general, Ki-67 and p53 are highly expressed in SRGC cells [12]. Ki-67 is an antigen that corresponds to a nuclear nonhistone protein expressed by cells in the proliferative phases [13]. Ki-67 labeling is relatively low in normal gastric epithelium (<15%), while Ki-67 labeling of primary SRGC is high [12]. p53 is a nuclear protein thought to be involved in the control of the cell cycle, apoptosis, and maintenance of genomic stability [14]. The immunohistochemical demonstration of p53 highly suggests a p53 gene mutation and malignant potential of tumors [14]. While normal epithelium never expresses p53, p53 is highly expressed in SRGC [12]. The expression levels of Ki-67 and p53 in early SRGCs are still unknown. Immunohistochemical analysis revealed Ki-67 and p53 mutations to be negative in this case, which might be the reason why it had not progressed to advanced gastric cancer for 15 months.

Recently, endoscopic submucosal dissection has been carefully applied in early SRGCs [15]. However, there are no specific guidelines for the endoscopic treatment of SRGCs. The differences in outcomes between early- and late-stage SRGC described above could reflect the aggressive resection strategies. Furthermore, it must be emphasized that forming satellite lesions is another characteristic of SRGC [4], for which extending endoscopic resection criteria is inappropriate. On the other hand, because there have been some reports that early SRGC carries a lower rate of LN metastasis, which is specifically related to recurrence of gastric cancer including SRGC, and better prognosis compared with advanced SRGC [7], it is possible that endoscopic resection can be performed on patients with small SRGC limited to the mucosa and with no LN metastasis or satellite lesions. CT indicated that there was a risk of serosal invasion of the gastric body, so we judged that laparoscopy-assisted distal gastrectomy, rather than endoscopic resection, was the most suitable treatment option to resect this gastric tumor completely. If serosal invasion had not been suspected, it might have been appropriate to treat this case with endoscopic resection such as endoscopic mucosal resection or endoscopic submucosal dissection. The surveillance of LN metastasis in addition to other organs and the residual stomach for primary SRGC is particularly important after endoscopic resection or surgery. Moreover, both endoscopic follow-up and biopsy are necessary to find SRGCs at early stages before diffuse infiltration can occur.
